# Commentary: Not in the drug, not in the brain: causality in psychedelic experiences from an enactive perspective

**DOI:** 10.3389/fpsyg.2023.1217108

**Published:** 2023-06-22

**Authors:** Ignacio Cea

**Affiliations:** ^1^Center for Research, Innovation and Creation, Temuco Catholic University, Temuco, Chile; ^2^Faculty of Religious Sciences and Philosophy, Temuco Catholic University, Temuco, Chile

**Keywords:** psychedelics, brain-consciousness relation, causal efficacy of consciousness, dynamic co-emergence, circular causality, interlevel causation, affect, enaction

## Introduction

I welcome with great enthusiasm Meling and Scheidegger's (2023; henceforth “M&S”) timely contribution to advance an enactive approach to psychedelic therapy, especially to the complex causality involved. Their two main research questions concerned: (i) the causal interaction between the psychedelic molecule and brain activity; and (ii) the causal interaction between brain activity and the psychedelic experience. While I largely agree with and celebrate much of what is proposed by M&S, especially their employment of key enactive concepts to advance our understanding of the first research question, in the following, I will present some worries regarding their answers to the second. Although I agree that there is probably a two-way reciprocal relationship between neural activity and experience, I have several points of contention regarding M&S's proposal. My hope is to stimulate discussion on M&S's important contribution, and to help advance a much-needed enactive science of psychedelics.

## Brain activity and psychedelic experience: dynamic co-emergence and circular causality

A concept that figures prominently in M&S's account of the relationship between brain activity and the psychedelic experience is *dynamic co-emergence* (henceforth “DCE”). A first worry is that DCE applies to the relationship between autonomous wholes and their parts (Thompson, 2007), but it is not clear that this mereological relationship holds for consciousness and brain activity. Arguably, the *parts* of a given psychedelic experience taken as a *whole* during certain time intervals (e.g., the experience of being dissolved into a cosmic unity), are *phenomenal parts* (e.g., feeling united to something greater, one's sense of self being disrupted, accompanying visual images, sounds, bodily sensations, thoughts, etc.), rather than local neural activity. Additionally, regarding the latter (i.e., *neural parts*), the corresponding whole is more plausibly a *neural whole*, i.e., a global brain activity such as interhemispheric synchronic gamma oscillations, rather than the experience itself.

A second issue is that M&S's treatment of DCE suggests that it is equivalent to circular causality, characterizing both in terms of global-to-local and local-to-global *determination*. However, they are related but distinct notions. While DCE is meant to describe the reciprocal, *constitutive* relationship between parts and wholes in autonomous systems (Thompson, 2007), circular causality characterizes the reciprocal but *causal* relationship between them (Haken, 1983; Kelso, 2021). While the difference between constitution and causation is a matter of ongoing debate (Aizawa, 2014; Kirchhoff, 2015), at least for a matter of theoretical clarity and to guide future research, they should be more clearly differentiated.

Third, I worry that the notion of DCE is currently too obscure to incentivize further psychedelic research from an enactive perspective. In contrast to circular causality, it is not obvious what DCE really amounts to. Thompson writes that “in an autonomous system… parts do not exist in advance, prior to the whole, as independent entities… part and whole co-emerge and mutually specify each other” (Thompson, 2007, p. 65). Of course, there is a sense in which this is certainly the case: a defining feature of *autopoietic* autonomous systems (e.g., a cell) is that its components are produced by the network of mutually enabling processes that constitute the system, and where global topological constraints play a key role (Maturana and Varela, 1980). Hence, there is a sense in which a protein molecule produced inside the cell may be said to have “emerged from the whole” or be “specified by the whole”. However, when applied to a brain network, it is far from obvious how to make sense of DCE. While it seems very plausible that a neuron *behaves* differently depending on whether it is part of system A rather than system B (i.e., an instance of global-to-local causality), it seems less plausible to hold that a neuron emerges from or is constitutively specified by the neural system it belongs to. Intuitively, a neuron remains being a neuron even if it were hypothetically isolated before being incorporated into, or after being separated from, a larger neural system, as long as it can remain potentially functional and structurally intact.

Fourth, in order to advance an enactive psychedelic science, circular causality should be formalized to make it a scientifically useful tool. To the best of my knowledge, the mathematical, dynamical approaches to circular causality that are most close to the enactive approach are the ones from Haken (1983) and Kelso (2021). Nonetheless, close attention should also be paid to formal accounts of causal emergence and downward causation from complexity science and information theory (Hoel et al., 2016; Mediano et al., 2022). Without an enactive, formal account of circular causality in psychedelic experience, M&S hardly improve the pluralistic view of causation and provide an “account of how biochemical, neural, and experiential processes affect each other through local-to-global and global-to-local determination” (Meling and Scheidegger, 2023, p. 9).

Fifth, as a relation between parts/local and wholes/global activity, in contrast to what is suggested by M&S, circular causality would be more straightforwardly involved in the relationship between the psychedelic molecule and brain activity, rather than between brain activity and the psychedelic experience. In the absence of sound reasons to consider the relationship between brain activity and conscious experiences as mereological, alternative ways to understand their causal relation should be looked for.

Finally, instead of focusing mostly on “psychedelic experiential cognitive acts” (Meling and Scheidegger, 2023, p. 9) involved in mystical-type experiences, future enactive research may concentrate also on the dynamics of the *affective* experience under psychedelics and its causal influence on the associated emotional-somatic changes. Experiencing an *emotional breakthrough* in the psychedelic session has also been validated as a strong mediator of subsequent mental health benefits (Roseman et al., 2019). Hence, an important theoretical foundation for an enactive psychedelic science would be the enactive approach to affectivity (Varela and Depraz, 2005; Colombetti, 2014). Importantly, the affective experiential dimension would have its primary locus in what Thompson and Varela (2001) called the *organismic regulation* cycle, and therefore, psychedelic-induced changes in the subject's primordial feeling of being alive or *continuous organismic sentience* (Cea and Martínez-Pernía, 2023) may have a key causal explanatory role to play.

## Author contributions

The author confirms being the sole contributor of this work and has approved it for publication.
